# 

**Published:** 1883-04

**Authors:** 


ORIGINAL COMMUNICATIONS:
Medical Clinic. Alfred L. Loomis, A.M., M.D. .... 193
Caries of Human Teeth. Frank Abbott, M.D. .... 198
Keeping the Teeth Clean. C. E. Francis, D.D.S., M.D.S. .... 204

ORIGINAL TRANSLATIONS AND ABSTRACTS:
An Operation for Pyonephrosis .... 206
Fungi of Tooth Caries .... 210

SELECTIONS:
Extirpation of Cancer of the Face, Involving Inferior Maxillary Bone, Floor of the Mouth (Right Side), Submaxillary and Parotid Glands .... 210
Gold *vs.* Amalgam .... 213
Koch's Bacillus and Its Lessons .... 215
Vomiting of Pregnancy .... 218
The New Anatomy Act .... 219

EDITORIAL:
Resignation .... 221
"A New Anæsthetic" .... 222
Dental Pathology .... 222
One Result of Koch's Investigations .... 222
Pain Obtunders .... 223
The Independent Practitioner .... 224
A Dangerous Remedy .... 225
Publisher's Notice .... 225

CURRENT NEWS AND OPINION:
Married .... 225
A Good Thing Well Bestowed .... 226
American Medical Association .... 226
American Dentists in Europe .... 226
Dental Society of the State of New York .... 227
Northern Ohio Dental Association .... 227
Georgia State Dental Society .... 228
Mad River Dental Society .... 228
The New Departure of the Association of American Medical Editors .... 228

BIBLIOGRAPHICAL .... 229

OBITUARY. W. H. Van Buren, M.D., LL.D., 231; Dr. W. H. Goddard .... 233

EXTRACTS:
Why is Chloroform so Well Borne in Midwifery? .... 234
A Case of Tumor .... 234
Dr. Beard on Herbert Spencer .... 235
A New Idea for the Spread of the Knowledge of Medical Ethics .... 236
Honest and True New York Doctors .... 236
The Micro-Organism of Whooping-Cough .... 237
Treatment of Ulcers .... 237
Fangs of the Rattlesnake .... 237
Statistics of Symphysiotomy .... 238
The Bacillus of Whooping-Cough .... 238

POPULAR SCIENCE DEPARTMENT:
The Medical Use of Music .... 240
Air Germs at Different Seasons .... 243
"The Missing Link" .... 244
Where the Backboned Animals Begin .... 245
Defective Calculations .... 246
False Perceptions .... 246

